# Simulators in urology resident’s training in retrograde intrarenal surgery

**DOI:** 10.1590/acb394724

**Published:** 2024-08-05

**Authors:** Luis Otávio Amaral Duarte Pinto, Renata Cunha Silva, Helder Clay Fares dos Santos, Lívia Guerreiro de Barros Bentes, Mateus Itiro Tamazawskas Otake, Herick Pampolha Huet de Bacelar, Kátia Simone Kietzer

**Affiliations:** 1 Universidade do Estado do Pará – Laboratory of Morphophysiology Applied to Health – Belém (PA) – Brazil.; 2 Universidade do Estado do Pará – Department of Microsurgery – Experimental Surgery Laboratory - Belém (PA) – Brazil.

**Keywords:** Education, Medical, Simulation Training, Printing, Three-Dimensional, Nephrolithiasis, Urology

## Abstract

**Purpose:**

To evaluate the impact of simulators on the training of urology residents in retrograde intrarenal surgery (RIRS).

**Methods:**

The study involved training eight urology residents, using two artificial simulators; one developed by the Universidade Estadual do Pará, using three-dimensional printing technology, and the other one patented by the medical equipment manufacturer Boston Scientific The qualification of residents took place through a training course, consisting of an adaptation phase (S0), followed by three training sessions, with weekly breaks between them (S1, S2 and S3). Study members should carry out a RIRS in a standardized way, with step-by-step supervision by the evaluator using a checklist. The participants’ individual performance was verified through a theoretical assessment, before and after training (pre- and post-training), as well as by the score achieved in each session on a scale called global psychomotor skill score. In S3, residents performed an analysis of the performance and quality of the simulation, by completing the scale of student satisfaction and self confidence in learning (SSSCL).

**Results:**

At the end of the course, everyone was able to perform the procedure in accordance with the standard. The training provided a learning gain and a considerable improvement in skills and competencies in RIRS, with p < 0.05. SSSCL demonstrated positive feedback, with an overall approval rating of 96%.

**Conclusions:**

Artificial simulators proved to be excellent auxiliary tools in the training of urology residents in RIRS.

## Introduction

Urinary lithiasis is a cosmopolitan, relapsing disease that causes high morbidity and high social cost. It is considered by many authors to be an important public health problem worldwide^1^. In Brazil, despite the scarcity of official data, in practice, there is a considerable number of patients with urinary stones, which evolves over years into serious conditions, such as sepsis and chronic renal failure^2^.

Despite the severity of this disease, technological advances in medicine have allowed increasingly less invasive therapeutic procedures, with retrograde intrarenal surgery (RIRS) currently considered the gold standard for most cases of kidney lithiasis^3^.

RIRS is a surgical procedure that consists of the use of a flexible endoscopic device, introduced through the patient’s urethra and capable of managing kidney stones in the most diverse positions of the urinary tract. A laser fiber can be connected to the equipment, allowing the stones to be fragmented into smaller pieces that can be extracted, without the need to cut the patient^4^.

This surgery is usually performed by urologists. To qualify for this medical specialty, health professionals in Brazil must have a degree in medicine and two medical residencies (general surgery and urology), totaling approximately 11 years of studies to enable them to treat, clinically or surgically, diseases of the genitourinary system^5^.

The majority of Brazilian urologists practice through a medical residency program (PRM), linked to the Unified Health System (SUS). Unfortunately, the learning of urology residents at SUS has been the target of criticism from them and from health education entities in the country^6^. The lack of investment in teaching hospitals has not kept pace with the growth of innovative technologies for the treatment of urological diseases. Therefore, many residents have deficiencies in their training, having poor contact with procedures considered essential nowadays for good practice in this specialty, as it is the case with endoscopic surgeries for the management of urinary lithiasis^7^.

Some health education institutions (HEIs) have been looking for alternatives to alleviate the difficulties encountered in training these professionals^8^. In this context, the use of simulators, more specifically synthetic training models, has been gaining increasing prominence as a complementary method in training surgical specialties^9^. Among the various advantages that involve the use of artificial simulators, in a healthcare teaching and learning environment, the following stand out:

- The possibility of developing training with varying degrees of difficulty;
- The opportunity to acquire surgical skills and competencies, without the stress of putting the patient at risk;
- Repeating the activity as many times as necessary for training, without the fear of making mistakes;
- Avoiding the unnecessary use of laboratory animals, which are not always present in all HEIs, and with increasingly more standards and laws rigid for use in an academic environment^10^.

One of the main reasons for boosting simulation, as an auxiliary tool in the teaching-learning binomial, is due to the improvement and popularization of three-dimensional printing technology, making it possible to create, in an increasingly realistic way, the most diverse organs of the human body, as well as the imitation of the operating field of surgical procedures considered to be extremely complex^11^. In urology, there are already publications on the use of simulators for learning robotic surgeries, video laparoscopic surgeries, and microsurgeries, among others^12^; however, the literature is still very scarce regarding the use of simulators in the training of endoscopic surgeries for the treatment of urinary lithiasis, such as RIRS.

Therefore, observing this lack of training for residents in our country, as well as the increasingly common use of simulation as a complementary teaching and learning tool, this study aimed to evaluate the impact of using simulators on resident training of urology at RIRS.

## Methods

### Ethical aspects

The study was developed at the Laboratory of Morphophysiology Applied to Health and the Laboratory of Experimental Surgery, at the Universidade Estadual do Pará (UEPA), complying with all ethical standards for research involving human beings, with its completion approved by the Research Ethics Committee of the institution, by obtaining a Certificate of Presentation of Ethical Appreciation number 48382121.9.0000.5174.

### Training in retrograde intrarenal surgery

The training of urology residents in the endoscopic treatment of urinary lithiasis occurred through the development of a training course offered by the Laboratory of Morphophysiology Applied to Health at UEPA, in partnership with the medical device manufacturing company Boston Scientific The sample consisted of eight urology residents, enrolled in two medical residency programs in the state of Pará, in the Amazon region of Brazil. All participants received theoretical guidance and training material two weeks before the start of the training, consisting of updated articles on the topic.

The course consisted of an adaptation phase (S0), followed by three training sessions, with weekly breaks between them (S1, S2 and S3, respectively). The setting (S0) consisted of a theoretical class, followed by practice involving basic notions about equipment management and the initial presentation to residents about how the simulation works. In order to verify the participants’ initial understanding of the topic, a theoretical assessment was carried out at the beginning of the setting (S0), through a test, containing 10 multiple-choice questions, with five alternatives each, which was called a pre-test. This assessment was repeated at the end of the training, to measure the level of learning acquired throughout the course, which became known as post-test.

**Figure 1:**
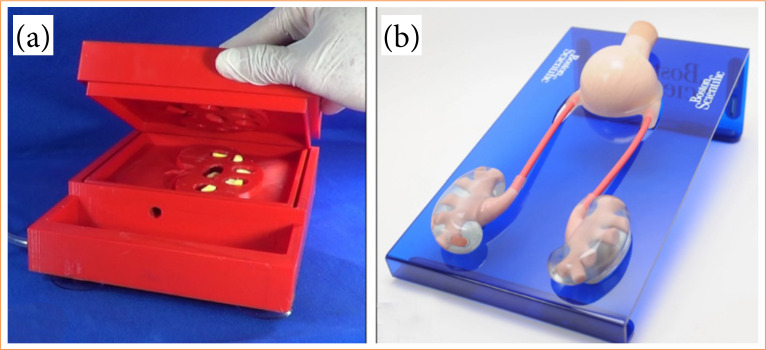
Training models. **(a)** Simulator made using three-dimensional printing for retrograde intrarenal surgery training. **(b)** High-fidelity simulator for retrograde intrarenal surgery training.

Source: (a) Pinto et al.^13^. (b) http://pulsemdm.com 14.

The following sessions were eminently practical, with training in RIRS, in which two artificial simulators were used, one medium and the other high fidelity. The medium fidelity simulator was used in the initial phase of the course (setting and S1). It was developed by UEPA and is presented in details, and duly validated by expert judges, in a previous publication^13^. It was created through three-dimensional printing, with polylactic acid (PLA) filaments, and basically consists of a training box that mimics part of the urinary system of an adult individual, having inside the anatomical shape of a kidney, containing a proximal portion of the ureter, pelvis and upper, middle and lower renal calyces (Fig. 1).

For the subsequent sessions (S2 and S3), a more realistic, high-fidelity simulator was used, patented by the company Boston Scientific and developed specifically for training in RIRS. It has the shape of a complete adult urinary system, containing a bladder, kidneys, and ureters, basically made of silicone and latex, fixed under acrylic support (Fig. 1). At the lower pole of each kidney, there is a cap, that can be removed to be introduced artificial urinary stones, composed of wax and commercial paraffin with a yellowish pigment, in a rounded shape.

**Figure 2:**
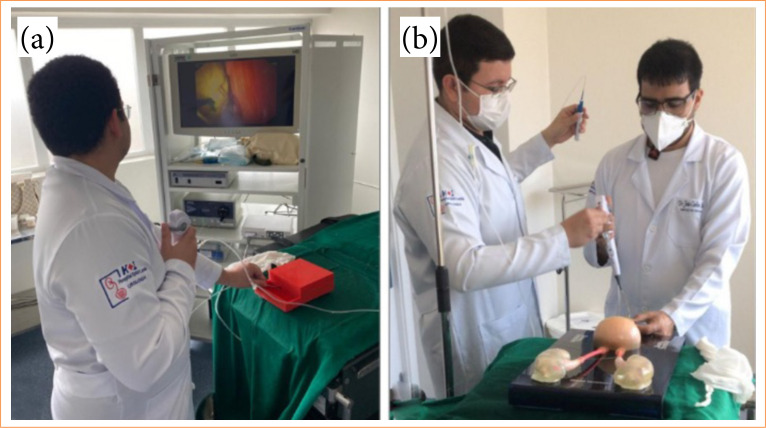
Training sessions. **(a)** Medium fidelity model. **(b)** High fidelity model.

Source: Elaborated by the authors.

The training sessions were carried out in pairs and lasted approximately 1 hour. Each resident was required to perform a flexible leisure ureterolithotripsy procedure in the experimental models (Fig. 2). The operation was standardized in stages, and the step-by-step execution was followed using a checklist (Table 1). At the end of each simulation, there was a moment of feedback, in which the evaluator highlighted the positive points presented by the participant and reinforced the points that needed to be improved, for better performance in the next sessions. It was expected that, by the end of the course, all residents would be able to perform a RIRS, within the proposed time and following all standardization steps.

**Table 1:** Retrograde intrarenal surgery standardization checklist.

Flexible ureteroscopy exercise		
Task description	Correct	Incorrect or incomplete
Navigation of all calyces		
Locating the stone(s)		
Use the basket to reposit the stone(s) from the lower calyx to the upper calyces*		
**Flexible ureteroscopy score (total number of correct tasks) — Maximum score: 3 points**		
**Laser Lithotripsy Exercise: Total time |Lithotripsy time**		
**Task description**	**Correct**	**Incorrect or** **incomplete**
Ureteroscope advancement		
Endoscopic laser fiber advancement		
Lithotripsy / fragmentation of the stone(s)		
Endoscopic basket introduction		
Extraction of calculation(s) (only a fragment)		
**Lithotripsy score (total number of correct tasks) — Maximum score: 5 points**		
**Checklist total (Maximum score: 8 points)**		

* If the task is correct, write down:

attempts with the basket (min. 1); accidental falling of the stone (min. 0). Source: Elaborated by the authors.

The residents’ individual performance was quantified by the researcher, at the end of each session, using an assessment scale called global psychomotor skill score (GPSS), consisting of seven aspects to be verified, with the score varying from 1 to 5 for each item^15^ (Table 2).

**Table 2:** Global psychomotor skill score (GPSS).

Respect for tissue	Frequently used unnecessary force or pushed scope into mucosa (white-out) or lost tunnel vision 1	2	Careful handling of tissues, but occasionally pushed scope into mucosa (whiteout) or lost tunnel vision 3	4	Consistently handled tissues with minimal force & maintained 360º (tunnel vision) of ureter during passage of scope 5
Time & motion	Many unnecessary moves 1	2	Efficient time/movements but some unnecessary moves 3	4	Clear economy of movement & maximum efficiency 5
Handling scope	Awkward & tentative moves and use of inappropriate instruments 1	2	Competent use of instrument, but occasional awkward moves 3	4	Fluid moves with instruments and no awkwardness 5
Knowledge of instrument	Frequently used wrong or inappropriate instruments 1	2	Mostly used appropriate instruments 3	4	Obviously familiar with and used all appropriate instruments 5
Flow of operations	Frequently stopped operating and seemed unsure 1	2	Had forward planning, but progressed tentatively at times 3	4	Planned operation well and had effortless flow of all moves 5
Use of assistants	Poorly placed or failed to use assistants 1	2	Used assistants well most of the time 3	4	Used assistants to best advantage all the time 5
Knowledge of speficic procedure	Needed specific instructions at all steps 1	2	Knew steps, but needed several instructions 3	4	Performed entire procedure without any instruction 5
**Add together all circled numbers for TOTAL GPSS SCORE: _____________________________**					

Source: Argun et al.^15^.

At the end of the training course (S3), residents also carried out an assessment of the quality of the simulation, as well as the learning acquired, by filling out a questionnaire, on a 5-point Likert scale, entitled scale of student satisfaction and self-confidence in learning (SSSCL)^16^ (Table 3), which covers 13 statements, divided into two domains: satisfaction with current learning (containing five statements), and self-confidence in learning (with the remaining eight statements). All sentences allow the following response possibilities: completely disagree (1 point), partially disagree (2 points), neither agree nor disagree (3 points), partially agree (4 points), and completely agree (5 points).

**Table 3:** Scale of student satisfaction and self confidence in learning.

Assessment
Satisfaction with current learning
1. The teaching methods used in this simulation were useful and effective.
2. The simulation provided me with a variety of teaching materials and activities to further my learning of the medical-surgical curriculum.
3. I liked the way the instructor taught through the simulation.
4. The teaching materials used in this simulation were motivating and enabled learning.
5. The way the instructor taught through simulation was suitable for the way I learned.
**Self-confidence in learning**
6. I am confident that I have mastered the content of the simulation activity that my instructor presented to me.
7. I am confident that this simulation included the content necessary to master the medical-surgical curriculum.
8. I am confident that I am developing skills and gaining the knowledge necessary from this simulation to perform the procedures in a real environment.
9. My instructor used useful resources to teach and deliver the simulation.
10. It is my responsibility as a student to learn what I need to perform the simulation activities effectively.
11. I know how to get help when I don't understand the concepts covered in the simulation.
12. I know how to use simulation activities to learn skills.
13. It is the instructor's responsibility to guide me on what I need to learn about the topic involved in carrying out the simulation.

Source: Almeida et al.^16^.

### Statistical analysis

The data were analyzed using the BioEstat 5.4 program. Initially, the results obtained were verified using the Shapiro-Wilk’s normality test. For parametric data comparison, the analysis of variance and paired Student’s t-test were The value of *p* ≤ 0.05. To analyze the SSSCL, the scores obtained in each of the 13 statements were calculated, as well as their averages in each domain, with the results expressed as average ± standard deviation. To study the reliability and internal consistency of the questionnaire, the Cronbach’s alpha index was used, with coefficient values above 0.75 being considered significant.

## Results

Tables 4 and 5 show the comparison of the assessment scores applied to the participants, before and after the training (pre- and post-test). Analyzing the values, it can be seen that the course contributed to theoretical learning in RIRS, given the better performance presented by all residents in the post-test, with the average varying from 6.0 ± 1.1 to approximately 8.0 ± 1.0 at the end of the course (*p* = 0.0010). In Table 4, it is possible to identify that all residents, by the end of the training, managed to reach the maximum score of 8 on the checklist, being able to perform the RIRS in accordance with the previously established standardization.

**Table 4:** Score obtained in the checklist.

Measures	Checklist*		
	Session 1	Session 2	Session 3
Minimum	3.0	6.0	8.0
Maximum	6.0	8.0	8.0
Median	5.0	8.0	8.0
Mean	4.8	7.4	8.0
± Standard deviation	± 1.0	± 0.9	± 0.0

* *p* < 0.0001;

one-way analysis of variance test. Source: Elaborated by the authors.

**Table 5:** Score obtained in the pre- and post-test theoretical assessment.

Measures	Assessment*	
	Pre-test	Post-test
Minimum	4.0	6.0
Maximum	7.0	9.0
Median	6.0	8.0
Mean	6.0	7.8
± Standard deviation	± 1.1	± 1.0

* *p* = 0.0010;

* tudent’s t-test for related samples.

Source: Elaborated by the authors.

**Figure 3:**
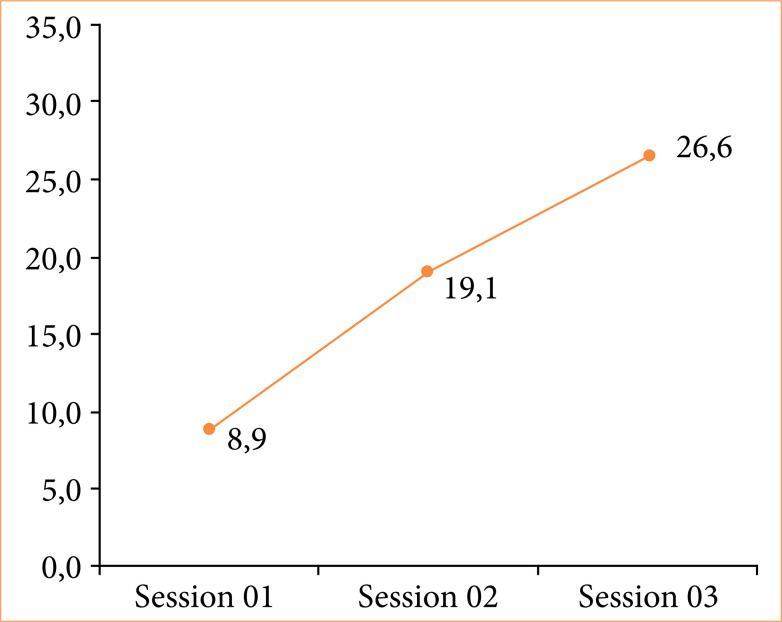
Average global psychomotor skill score throughout the training sessions.

Figure 3 consists of a graphic representation of the gain in skills and competencies in RIRS, through the observation of the average score achieved in the GPSS by urology residents during training. In this image, a progressive and significant increase, from a statistical point of view, can be seen in the score throughout the weekly sessions, with its values reaching a peak of 26.6 points on average in S3 (*p* < 0.0001).

*p < 0.0001; one-way analysis of variance test. Source: Elaborated by the authors.

Table 6, finally, expresses the SSSCL score, which was applied to participants at the end of the course. The analysis constructed through this allows us to ratify the residents’ satisfaction with the learning obtained during this training period, making it possible to verify an overall approval of 96%, with a Cronbach’s alpha coefficient of 81%.

**Table 6:** Residents’ assessment, using the scale of student self-confidence in learning, of retrograde intrarenal surgery training.

Assessment domains	Assessment		Cronbach’s index
	Score	% Approval	
Learning	199	99.5	0.790
Self-confidence	300	93.8	0.840
**General evaluation**	**499**	**96.0**	**0.815**

Source: Elaborated by the authors.

## Discussion

Medical residency is considered, by the Ministry of Health, the gold standard of specialization courses in medicine^17^. Its main objective is to improve the professional competence acquired during graduation. Such refinement includes training in some medical specialty; the progressive acquisition of responsibilities for medical acts; the development of the capacity for initiative, judgment, and evaluation; the internalization of ethical precepts and norms, and the development of a critical spirit^18^.

According to the competency matrix for PRM in urology in Brazil, prepared in 2018 by the Ministry of Education, in partnership with the Brazilian Society of Urology, residents of this specialty, at the end of their training, must be able to understand pathophysiology, the diagnosis and clinical treatment of urinary lithiasis, as well as being prepared to carry out surgical treatment when indicated^19^.

Guidelines from both the American Urological Association and the European Association of Urology have recommended RIRS as the first option in the management of kidney stones and proximal ureters measuring up to 2 cm, or larger than that, depending on the complexity of the case and the patient’s clinical condition^20^^,^ 21. Unfortunately, like most public educational institutions in Brazil, we highlight some gaps in the training of our residents, especially those procedures that depend on high-cost equipment (as is the case with RIRS). This is a problem that should not happen in a urology PRM located in the Amazon, a region that has a high prevalence of this disease, favored by environmental and sociocultural factors, which is considered an important cause of chronic renal failure^22^.

This study is a continuation of the research developed by the UEPA, to find alternatives to the gaps in the training of our specialists. In recent years, we have seen an increasing number of publications valuing simulation as an auxiliary tool in the training of surgical areas, which was a motivating factor to start a line of research in 2022 that culminated in the development of an experimental model, in three-dimensional printing, for RIRS training.

According to Antoniou et al.^23^, the benefits of simulated training are: low cost, given the lack of concern with sterilization, in addition to allowing the reuse of materials; the possibility of repeated training several times; the opportunity to make mistakes and learn from mistakes, without the risk of iatrogenic events; the practicality of reconciling the training of residents in the gaps that exist between their busy schedule of outpatient care, visits the infirmary and procedures in the surgical suite, among others. We believe that the training course implemented by UEPA managed to demonstrate not only these benefits, but also to identify in the study participants other indirect and intangible gains from simulation-based learning, referred to by some authors as “soft skills”, which comprise teamwork, mutual respect, effective communication, leadership, and other things^23^.

The initial idea was to use only the simulator developed by UEPA to train residents, but thanks to the partnership that was consolidated with the regional representative of the company Boston Scientific it was possible to add a high-fidelity simulator to the course, which guaranteed an upgrade in the quality of the training offered, allowing residents to achieve all the objectives recommended by Crouch et al.^24^, which are:

- Use of a specific equipment;
- Performance of certain manual movements;
- Recognition and familiarity with anatomical locations; replication of a surgical procedure in its entirety.

The literature is very dubious regarding the standard format of RIRS training courses, with most studies varying between one and five training days^23^. In our reality, five sessions would make training unfeasible, due to the rental costs of some equipment, such as the laser generator and the flexible ureteroscope. In turn, we do not recommend training courses with just one practical training session, as we believe that this is insufficient for the acquisition of skills and competencies in RIRS. In our study, by way of illustration, no participant managed to reach the 8 points of the checklist, referring to the standardization of the procedure in S1.

In this work, we chose to develop a course containing three sessions, with weekly breaks between them, which we consider quite satisfactory, as it allowed the progressive gain of skills and competence, with all residents able, until the last session, to carry out a standardized RIRS. We believe that sessions with weekly breaks are more productive, compared to intensive courses on busy days. The classic study by Moulton et al.^25^ suggests that, during training intervals, different regions of the brain become activated, each is considered necessary for permanent retention of surgical skills and competencies. The fact of searching your memory for key aspects of the skill being learned helps to solidify this skill more deeply in memory^25^.

The literature has demonstrated several ways of evaluating performance during training sessions, which involve the use of checklists, scales, and performance measurement using movement sensors, among others, with a clear preference for global rating scales (GRS)^26^. For Ghanem et al.^26^, the use of GRS in these assessments has the main objective of increasing the objectivity and quantification of performance, reducing the subjective effect of this assessment. Furthermore, another highly valuable aspect of this tool is that it allows residents who participated in the study to monitor their progress, facilitating during the debriefing the identification of points that need to be improved for the next training sessions^26^. In this study, we decided to use the GPSS, a variant of a GRS called objective structured assessment of technical skill (OSATS), which is the scale most currently used to quantify performance in simulated activities^15^. The GPSS was created in 2015 by Argun et al.^15^ and was chosen because it was developed precisely to measure skills and competencies in endourological surgical procedures, with its statements being adapted and more consistent with what was practiced during our training in RIRS.

The present manuscript involved the training of all urology residents, enrolled in the PRM of the state of Pará, in the endoscopic management of renal lithiasis, with the results achieved proving to be quite encouraging, about learning and gaining skills in competencies, with the RIRS simulators. This was verified through the significant increase, from a statistical point of view, in the post-test scores, compared to the pre-test, as well as the progressive growth in the checklist and GPSS scores. It is important to highlight that the findings included in this study are by most publications on the topic^10^^,^ 27. Hussain et al.^27^, for example, validated a low-fidelity simulator for training urology residents at RIRS, through a one-day training course. Participants were evaluated using the OSATS, reaching an average score of 24 ± 4.5 at the end of the course. In turn, Soria et al.^10^ carried out a two-day hybrid simulation course, using a high-fidelity experimental model, together with practice in pigs. In their work, participants showed a jump in the OSATS score, from the first to the second session, from 11.85 ± 0.43 to 27.22 ± 0.52, with p < 0.0001.

The implementation of the SSSCL aimed to seek feedback from urology residents about their perception, as well as the degree of satisfaction, about the learning obtained at the end of the training course in RIRS. This tool was used in this research because we believe that the resident’s well-being and the self-confidence acquired through learning are important constructs in the working environment and knowing how to measure them can provide us with valuable information for structuring teaching plans. The feedback obtained from SSSCL was very encouraging, with an overall satisfaction rate of 96%, serving as a stimulus to continue this line of research at the university.

The main negative point observed in this research lies in the fact that, despite the increasing use of training in medical-surgical specialties, through synthetic simulators, the literature still recommends, as a gold standard, training using cadavers or live models^28^. Even though there are similarities, most artificial high-fidelity simulators cannot, in fact, mimic all the peculiarities of the human urinary system, as well as the perfect training of all stages of surgeries.

Despite these drawbacks, the use of synthetic models has been encouraged by the main international urology teaching entities. Ahmed et al.^29^ published the European Association of Urology guidelines for training urology residents in urolithiasis, which reinforces that even low-fidelity models allow basic and intermediate-level training, as well as the acquisition of technical skills^29^. Our institution has been adhering to the three Rs policy, involving animal experimentation (reduction in the number of animals used; replacement with other experimental models, and refinement in the care of guinea pigs). Nowadays, with animal practice restricted to a few HEIs and with increasing pressure from society to reduce experimentation with live models, investing in artificial simulation seems to be an excellent option.

We expect that this study can contribute to the search for improvements in the standardization of training courses in RIRS, as well as serve as a stimulus for the creation of experimental models in three-dimensional printing, increasingly realistic, with cost optimization, and that can allow reliable training, not only for all stages of RIRS, but also for other endoscopic procedures, such as: rigid ureterolithotripsy, cystolithotripsy, percutaneous nephrolithotripsy, among others.

## Conclusion

Artificial simulators proved to be great auxiliary tools in the training of urology residents in RIRS.

## Data Availability

All data sets were generated or analyzed in the current study.

## References

[1] Hill AJ, Basourakos SP, Lewicki P, Wu X, Arenas-Gallo C, Chuang D, Bodner D, Jaeger I, Nevo A, Zell M, Markt SC, Eisner BH, Shoag JE (2022). Incidence of Kidney Stones in the United States: The Continuous National Health and Nutrition Examination Survey. J Urol.

[2] Silva SF, Silva SL, Campos H de H, Daher Ede F, Silva CA (2011). Demographic, clinical, and laboratory data of patients with urinary lithiasis in Fortaleza, Ceará. J Bras Nefrol.

[3] García Fadrique, Budía A, Climent L, Palmero JL, Morera J, Galán JA, Gil J, Montoya D, García P, Montoliu A, Pastor F, Gallego D (2019). Adherence to the European Association of Urology Guidelines Regarding the Therapeutic Indications for the Treatment of Urinary Lithiasis: A Spanish Multicenter Study. Urol Int.

[4] Kozyrakis DG, Kratiras ZK, Perikleous SK, Zarkadas AP, Chatzistamoy SE, Karagiannis DK, Solinis IT (2019). How Effective Is Retrograde Semirigid and Flexible Ureteroscopic Lithotripsy for the Treatment of Large Ureteral Stones Equal to or Greater than 15 mm? Results from a Single Center. Urol Int.

[5] Gorgen ARH, Diaz JO, da Silva AGT, Paludo A, de Oliveira RT, Tavares PM, Rosito TE (2021). The impact of the COVID-19 pandemic on urology practice, assistance, and residency training in a tertiary referral center in Brazil. Int Braz J Urol.

[6] Chioro A, Andreazza R, Furtado LAC, Araújo EC, Nasser MA, Cecílio LCO (2021). The contracting policy of teaching hospitals: what did actually change?. Ciên Saúde Colet.

[7] Prezotti JA, Henriques JVT, Favorito LA, Canalini AF, Machado MG, Brandão TBV, Barbosa AMV, Moromizato JKM, Anzolch KMJ, Fernandes RC, Rodrigues FRA, Bellucci CHS, Silva CS, Pompeo ACL, de Bessa J, Gomes CM (2021). Impact of COVID-19 on education, health and lifestyle behavior of Brazilian urology residents. Int Braz J Urol.

[8] Aditya I, Kwong JCC, Canil T, Lee JY, Goldenberg MG (2020). Current Educational Interventions for Improving Technical Skills of Urology Trainees in Endourological Procedures: A Systematic Review. J Endourol.

[9] Pinto LOAD, de Barros CAV, de Lima AB, Dos Santos DR, Bacelar HPH (2019). Portable model for vasectomy reversal training. Int Braz J Urol.

[10] Soria F, Morcillo E, Serrano A, Cansino R, Rioja J, Fernandez I, de la Cruz J, Van Cleynenbreugel B, Sanchez-Margallo FM (2015). Development and Validation of a Novel Skills Training Model for Retrograde Intrarenal Surgery. J Endourol.

[11] Sarikaya S, Dourado Meneses A, Cacciamani GE, Gómez Rivas J (2018). Future of Urology training. Arch Esp Urol.

[12] Smith B, Dasgupta P (2020). 3D printing technology and its role in urological training. World J Urol.

[13] Pinto LOAD, Sílva RC, Júnior HC, Bentes LG, Bacelar HP, Kietzer KS (2022). 3D printed simulator for training in flexible laser ureterolithotripsy. Rev Eletronica Acervo Saude.

[14] Medical Device Models (2024). Pulse Medical Demonstration Models.

[15] Argun OB, Chrouser K, Chauhan S, Monga M, Knudsen B, Box GN, Lee DI, Gettman MT, Poniatowski LH, Wang Q, Reihsen TE, Sweet RM (2015). Multi-Institutional Validation of an OSATS for the Assessment of Cystoscopic and Ureteroscopic Skills. J Urol.

[16] Almeida RG dos S, Mazzo A, Martins JCA, Baptista RCN, Girão FB, Mendes IAC (2015). Validation to Portuguese of the Scale of Student Satisfaction and Self-Confidence in Learning. Rev Latino-Am Enferm.

[17] Sassi AP, Seminotti EP, Paredes EA, Vieira MB (2020). The Professional Ideal in Medical Training. Rev Bras Educ Medica.

[18] Costa JB, Austrilino L, Medeiros ML (2021). Perceptions of resident doctors about the Pediatrics residency program at a public university hospital. Interface.

[19] MATRIZ DE COMPETÊNCIAS - UROLOGIA (2018). MATRIZ DE COMPETÊNCIAS - UROLOGIA.

[20] Skolarikos A, Neisius HJ, Petrik A, Somani B, Tailly T, Gambaro G (2023). Guidelines on Urolithiasis.

[21] Assimos D, Krambeck A, Miller NL, Monga M, Murad MH, Nelson CP, Pace KT, Pais VM, Pearle MS, Preminger GM, Razvi H, Shah O, Matlaga BR (2016). Surgical management of stones: American Urological Association/Endourological Society Guideline, part II. J Urol.

[22] Wagner CA (2021). Etiopathogenic factors of urolithiasis. Arch Esp Urol.

[23] Antoniou V, Gauhar V, Kallidonis P, Skolarikos A, Veneziano D, Liatsikos E, Somani BK (2023). Education and training evolution in urolithiasis: A perspective from European School of Urology. Asian J Urol.

[24] Crouch G, Wong G, Hong J, Varey A, Haddad R, Wang ZZ, Wykes J, Koutalistras N, Clark JR, Solomon M, Bannon P, McBride KE, Ch’ng S (2021). Validated specialty-specific models for multi-disciplinary microsurgery training laboratories: a systematic review. ANZ J Surg.

[25] Moulton CA, Dubrowski A, Macrae H, Graham B, Grober E, Reznick R (2006). Teaching surgical skills: what kind of practice makes perfect?: a randomized, controlled trial. Ann Surg.

[26] Ghanem A, Kearns M, Ballestín A, Froschauer S, Akelina Y, Shurey S, Legagneux J, Ramachandran S, Cozzolino S, Ramakrishnan V, Pafitanis G, Zakaria Y, Al-Maaytah K, Komatsu S, Kimata Y, Cifuentes I, Soucacos PN, Tos P, Myers S (2020). International microsurgery simulation society (IMSS) consensus statement on the minimum standards for a basic microsurgery course, requirements for a microsurgical anastomosis global rating scale and minimum thresholds for training. Injury.

[27] Hussain S, Rana RS, Ather MH (2021). Validation of a Bench-Top Training Model for Retrograde Intrarenal Surgery. Urol Int.

[28] Huri E, Skolarikos A, Tatar İ, Binbay M, Sofikerim M, Yuruk E, Karakan T, Sargon M, Demiryurek D, Miano R, Bagcioglu M, Ezer M, Cracco CM, Scoffone CM (2016). Simulation of RIRS in soft cadavers: a novel training model by the Cadaveric Research On Endourology Training (CRET) Study Group. World J Urol.

[29] Ahmed K, Patel S, Aydin A, Veneziano D, van Cleynenbreugel B, Gözen AS, Skolarikos A, Sietz C, Lahme S, Knoll T, Redorta JP, Somani BK, Sanguedolce F, Liatsikos E, Rassweiler J, Khan MS, Dasgupta P, Sarica K (2018). European Association of Urology Section of Urolithiasis (EULIS) Consensus Statement on Simulation, Training, and Assessment in Urolithiasis. Eur Urol Focus.

